# Supplementary material for: Intersectionality-Informed Sex/Gender-Sensitivity in Public Health Monitoring and Reporting (PHMR): A Case Study Assessing Stratification on an “Intersectional Gender-Score”
Source: Int J Environ Res Public Health. 2023 Jan 26;20(3):2220. doi: 10.3390/ijerph20032220 (PMC9916012; doi:10.3390/ijerph20032220)
Supplement: Supplementary file 1 [file ijerph-20-02220-s001.zip › ijerph-2147739-supplementary.pdf]

Table S1: Sensitivity analysis assessing the number of imbalanced categories of covariables (Standardized Mean Difference > 0.1) with Generalised Boosted Models (GBM) accounting for missingness with surrogate splits or multiple imputation prior to GBM testing 4-7 subgroups to stratify on the Intersectional Gender-Score.

	GBM with surrogate splits SMD cut-off 0.1				Multiple imputation prior to GBM SMD cut-off 0.1			
	4 Strata	5 Strata	6 Strata	7 Strata	4 Strata	5 Strata	6 Strata	7 Strata
Subgroup 1	4	5	7	6	4	5	5	6
Subgroup 2	0	1	0	1	0	0	0	2
Subgroup 3	0	0	0	1	1	0	0	0
Subgroup 4	15	0	0	0	13	0	0	0
Subgroup 5	-	10	7	0	-	12	7	1
Subgroup 6	-	-	12	8	-	-	11	10
Subgroup 7	-	-	-	15	-	-	-	7

Abbreviations: SMD - Standardized Mean Difference

Figure S1: Density plot - visual inspection of the distributions of the IG-Score for men and women

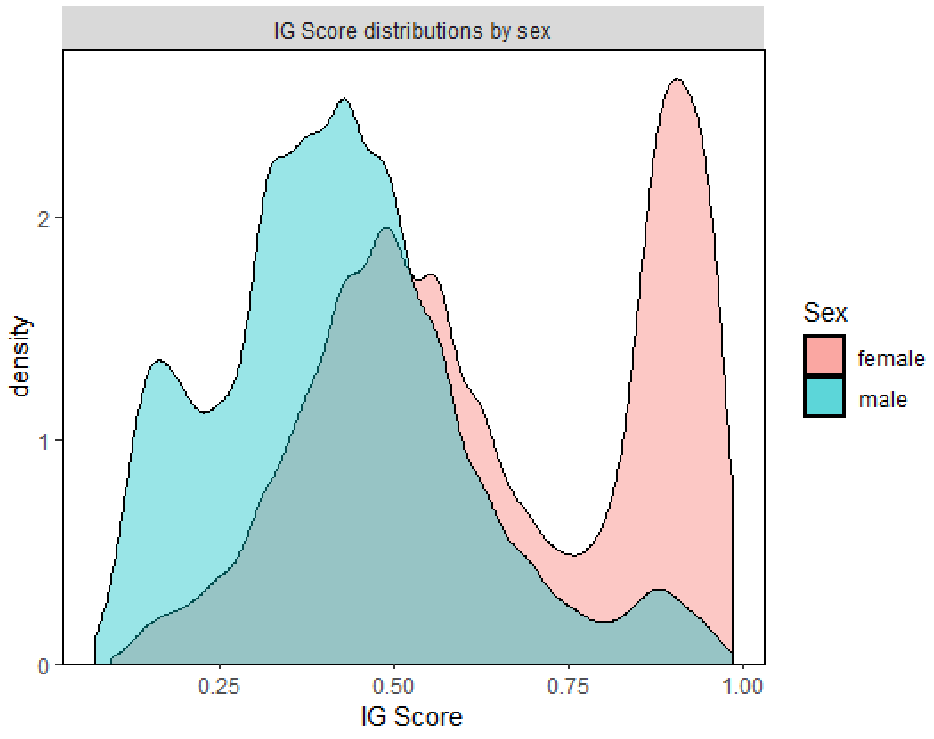

Table S2: Balance diagnostics of covariables between men and women in the total sample and the 5 subgroups after stratification on the Intersectional Gender-Score.

Variable	SMD Full sample	SMD Subgroup 1	SMD Subgroup 2	SMD Subgroup 3	SMD Subgroup 4	SMD Subgroup 5
**N**	23159	4645	4619	4662	4601	4632
**Age group**						
18-29 years	-0.06	0.03	0.03	0.00	-0.05	0.02
30-39 years	0.07	0.01	0.02	-0.03	0.01	0.05
40-49 years	0.04	0.05	-0.04	0.03	-0.02	0.08
50-59 years	0.00	-0.07	-0.08	0.01	0.08	-0.03
60-69 years	-0.03	0.00	0.02	-0.04	-0.02	-0.19
70-79 years	-0.04	-0.03	0.02	0.04	-0.02	0.12
80+	-0.01	-0.05	0.01	0.01	0.02	-0.04
**Employment status**						
Working and in education	0.02	-0.02	0.02	-0.04	-0.02	-0.07
full-time	-0.66	-0.01	0.00	0.01	0.00	-0.09
part-time	0.61	-0.01	-0.04	-0.06	0.07	-0.16
occasionally	0.20	-0.01	-0.01	-0.02	0.02	-0.03
education	-0.06	0.10	0.03	-0.01	0.01	0.04
retired	-0.03	-0.04	0.03	0.02	-0.02	0.01
maternity/parental leave	0.22	0.00	0.00	0.00	0.03	0.17
unemployed	-0.01	-0.01	-0.08	0.08	-0.07	0.04
housewife/ -husband	0.34	0.00	0.00	0.00	-0.06	0.14
other	-0.02	-0.06	-0.04	0.03	0.04	-
missing	-0.02	-0.04	-0.03	-0.01	0.04	-
**Occupational status**						
blue collar	-0.30	-0.19	-0.04	0.03	0.08	-0.12
white collar	0.16	0.12	-0.02	-0.01	-0.02	-0.06
civil servant	-0.03	0.02	0.00	0.00	-0.01	-0.04
freelance	-0.13	0.09	-0.02	-0.03	0.01	-0.04
helping family	0.03	0.00	-0.04	-0.03	0.04	0.06
apprenticeship	-0.05	0.07	0.03	-0.04	0.02	-0.02
else	0.13	-0.05	0.04	0.03	-0.04	0.17
missing	0.00	-0.06	-0.01	0.02	-0.01	0.05
**Education**						
high	-0.03	0.13	0.01	-0.02	-0.02	-0.14
middle	0.12	0.01	-0.02	-0.02	0.03	0.19
low	-0.09	-0.17	-0.02	0.05	0.01	0.02
else	0.01	0.03	0.03	0.00	-0.02	-0.08
missing	-0.03	0.03	-0.01	0.04	0.00	-0.06
**Partner in household**						
yes	-0.11	-0.06	-0.03	0.04	0.01	0.09
no	0.11	0.06	0.03	-0.04	-0.01	-0.09
missing	0.00	-0.05	0.01	0.02	0.01	-0.08
**Children <16 years in household**						

yes	0.05	-0.07	-0.11	0.01	0.06	0.22
no	-0.05	0.07	0.11	-0.01	-0.06	-0.22
**Migration background**						
none	-0.02	0.02	0.01	-0.01	0.01	-0.06
direct	0.02	-0.02	-0.03	0.02	0.00	0.02
indirect	0.00	-0.01	0.01	-0.01	-0.01	0.08
**Household language**						
German	0.02	0.05	0.02	0.00	0.02	-0.02
other	-0.02	-0.05	-0.02	0.00	-0.02	-0.02
missing	0.01	-0.01	-0.07	0.03	0.02	-0.06
**Feeling lonely**						
often	0.12	0.03	-0.06	0.06	-0.02	0.05
seldom/never	-0.12	-0.03	0.06	-0.06	0.02	-0.05
missing	-0.01	-0.02	-0.03	-0.01	0.01	0.05
**Disability**						
yes	-0.04	0.01	0.02	0.03	-0.07	-0.07
no	0.04	-0.06	-0.02	-0.03	0.07	0.07
missing	0.02	0.05	0.02	-0.02	0.00	-0.03
**Urbanity/Rurality**						
urban	0.02	-0.01	0.03	-0.01	-0.02	-0.01
rural	-0.02	0.01	-0.03	0.01	0.02	0.01
**Household help**						
yes, regularly	-0.01	0.07	0.00	0.03	-0.04	-0.06
yes, occasionally	0.00	-0.01	-0.03	0.02	0.02	0.05
no	0.01	-0.06	0.01	-0.04	0.02	0.04
missing	0.00	-0.03	-0.04	0.04	0.01	-

Abbreviations: SMD - Standardized Mean Difference

Table S3: Description of the total sample and the 5 subgroups defined by the Intersectional Gender-Score, stratified by men/women.

Variable	Full sample		Subgroup 1		Subgroup 2		Subgroup 3		Subgroup 4		Subgroup 5	
N	23159		4645		4619		4662		4601		4632	
	men	women	men	women	men	women	men	women	men	women	men	women
Sex % (n)	46.31 (10724)	53.69 (12435)	82.41 (3828)	17.59 (817)	61.07 (2821)	38.93 (1798)	50.15 (2338)	49.85 (2324)	30.75 (1415)	69.25 (3186)	6.95 (322)	93.05 (4310)
Age group % (n)												
18-29 years	19.96 (2141)	17.71 (2202)	8.20 (314)	9.06 (74)	25.20 (711)	26.42 (475)	33.88 (792)	33.99 (790)	21.77 (308)	19.77 (630)	4.97 (16)	5.41 (233)
30-39 years	15.47 (1659)	18.16 (2258)	17.32 (663)	17.87 (146)	18.61 (525)	19.30 (347)	10.95 (256)	9.98 (232)	9.19 (130)	9.51 (303)	26.40 (85)	28.54 (1230)
40-49 years	20.37 (2184)	22.18 (2758)	38.06 (1457)	40.64 (332)	5.35 (151)	4.56 (82)	9.41 (220)	10.15 (236)	17.74 (251)	17.01 (542)	32.61 (105)	36.33 (1566)
50-59 years	18.83 (2019)	18.71 (2326)	24.48 (937)	21.54 (176)	14.68 (414)	11.90 (214)	15.06 (352)	15.49 (360)	17.17 (243)	20.40 (650)	22.67 (73)	21.48 (926)
60-69 years	13.10 (1405)	12.25 (1523)	7.29 (279)	7.34 (60)	14.36 (405)	15.24 (274)	19.97 (467)	18.33 (426)	15.05 (213)	14.25 (454)	12.73 (41)	7.17 (309)
70-79 years	8.73 (936)	7.62 (948)	2.32 (89)	1.96 (16)	16.02 (452)	16.63 (299)	10.14 (237)	11.40 (265)	11.17 (158)	10.55 (336)	0.0 (0)	0.74 (32)
80+	3.54 (380)	3.38 (420)	2.32 (89)	1.59 (13)	5.78 (163)	5.95 (107)	0.60 (14)	0.65 (15)	7.92 (112)	8.51 (271)	0.62 (2)	0.32 (14)
Employment status % (n)												
Working and in education	3.08 (330)	3.46 (430)	0.03 (1)	0 (0)	2.02 (57)	2.34 (42)	6.46 (151)	5.46 (127)	8.41 (119)	7.94 (253)	0.62 (2)	0.19 (8)
full-time	54.82 (5879)	24.56 (3054)	90.99 (3483)	90.94 (743)	44.42 (1253)	44.77 (805)	36.01 (842)	35.54 (826)	21.13 (299)	21.22 (676)	0.62 (2)	0.09 (4)
part-time	4.18 (448)	24.41 (3036)	0.42 (16)	0.37 (3)	0.96 (27)	0.61 (11)	1.67 (39)	0.99 (23)	10.39 (147)	12.49 (398)	68.01 (219)	60.35 (2601)
occasionally	2.93 (314)	7.31 (909)	1.07 (41)	0.98 (8)	1.28 (36)	1.22 (22)	2.01 (47)	1.81 (42)	10.67 (151)	11.39 (363)	12.11 (39)	11.00 (474)
education	9.45 (1013)	7.71 (959)	1.85 (71)	3.43 (28)	14.25 (402)	15.29 (275)	18.91 (442)	18.37 (427)	6.93 (98)	7.09 (226)	0 (0)	0.07 (3)
retired	18.40 (1973)	17.19 (2137)	3.37 (129)	2.69 (22)	27.33 (771)	28.59 (514)	27.84 (651)	28.70 (667)	29.75 (421)	28.78 (917)	0.31 (1)	0.39 (17)
maternity/parental leave	0.12 (13)	2.77 (344)	0 (0)	0 (0)	0 (0)	0 (0)	0 (0)	0 (0)	0 (0)	0.03 (1)	4.04 (13)	7.96 (343)
unemployed	4.96 (532)	4.76 (592)	1.18 (45)	1.10 (9)	7.37 (208)	5.45 (98)	5.47 (128)	7.36 (171)	10.53 (149)	8.47 (270)	0.62 (2)	1.02 (44)
housewife/ -husband	0.48 (51)	6.60 (821)	0 (0)	0 (0)	0 (0)	0 (0)	0 (0)	0 (0)	0.49 (7)	0.16 (5)	13.66 (44)	18.93 (816)
other	0.49 (53)	0.34 (42)	0.47 (18)	0.12 (1)	0.78 (22)	0.44 (8)	0.34 (8)	0.56 (13)	0.35 (5)	0.63 (20)	0 (0)	0 (0)
missing	1.10 (118)	0.89 (11)	0.63 (24)	0.37 (3)	1.60 (45)	1.28 (23)	1.28 (30)	1.20 (28)	1.34 (19)	1.79 (57)	0 (0)	0 (0)

Occupational status<br>% (n)												
blue collar	17.67<br>(1895)	7.90<br>(982)	40.05<br>(1533)	31.09<br>(254)	6.03<br>(170)	5.17<br>(93)	2.27<br>(53)	2.71<br>(63)	7.77<br>(110)	9.98<br>(318)	9.01<br>(29)	5.89<br>(254)
white collar	35.22<br>(3777)	43.11<br>(5361)	37.49<br>(1435)	43.57<br>(356)	30.02<br>(847)	28.98<br>(521)	34.56<br>(808)	34.08<br>(792)	33.92<br>(480)	32.99<br>(1051)	64.29<br>(207)	61.28<br>(2641)
civil servant	4.36<br>(468)	3.79<br>(471)	4.91<br>(188)	5.26<br>(43)	5.21<br>(147)	5.28<br>(95)	3.25<br>(76)	3.27<br>(76)	2.97<br>(42)	2.86<br>(91)	4.66<br>(15)	3.85<br>(166)
freelance	6.95<br>(745)	3.98<br>(495)	10.71<br>(410)	13.59<br>(111)	5.99<br>(169)	5.51<br>(99)	3.93<br>(92)	3.40<br>(79)	4.88<br>(69)	5.02<br>(160)	1.55<br>(5)	1.07<br>(46)
helping family	0.09<br>(10)	0.20<br>(25)	0<br>(0)	0<br>(0)	0.07<br>(2)	0<br>(0)	0.21<br>(5)	0.09<br>(2)	0.21<br>(3)	0.44<br>(14)	0<br>(0)	0.21<br>(9)
apprenticeship	3.72<br>(399)	2.90<br>(360)	1.33<br>(51)	2.20<br>(18)	5.92<br>(167)	6.56<br>(118)	6.07<br>(142)	5.12<br>(119)	2.69<br>(38)	3.01<br>(96)	0.31<br>(1)	0.21<br>(9)
else	31.70<br>(3400)	37.84<br>(4705)	5.30<br>(203)	4.28<br>(35)	46.51<br>(1312)	48.28<br>(868)	39.44<br>(1156)	50.95<br>(1184)	46.93<br>(664)	45.17<br>(1439)	20.19<br>(65)	27.35<br>(1179)
missing	0.28<br>(30)	0.29<br>(36)	0.21<br>(8)	0<br>(0)	0.25<br>(7)	0.22<br>(4)	0.26<br>(6)	0.39<br>(9)	0.64<br>(9)	0.53<br>(17)	0<br>(0)	0.14<br>(6)
Education<br>% (n)												
high	31.11<br>(3336)	29.88<br>(3715)	28.50<br>(1091)	34.03<br>(278)	38.67<br>(1091)	39.27<br>(706)	28.87<br>(675)	27.80<br>(646)	25.65<br>(363)	24.83<br>(791)	36.02<br>(116)	30.03<br>(1294)
middle	24.56<br>(2634)	29.75<br>(3700)	22.20<br>(850)	22.52<br>(184)	15.99<br>(451)	15.52<br>(279)	35.50<br>(830)	34.04<br>(791)	29.89<br>(423)	31.14<br>(992)	24.84<br>(80)	33.74<br>(1454)
low	25.41<br>(2725)	21.76<br>(2706)	29.73<br>(1138)	22.28<br>(182)	26.02<br>(734)	25.31<br>(455)	18.82<br>(440)	20.65<br>(480)	25.30<br>(358)	25.64<br>(817)	17.08<br>(55)	17.91<br>(772)
else	14.42<br>(1546)	14.74<br>(1833)	16.27<br>(623)	17.38<br>(142)	12.44<br>(351)	13.40<br>(241)	12.66<br>(296)	12.48<br>(290)	15.19<br>(215)	14.47<br>(461)	18.94<br>(61)	16.22<br>(699)
missing	4.50<br>(483)	3.87<br>(481)	3.29<br>(126)	3.79<br>(31)	6.88<br>(194)	6.51<br>(117)	4.15<br>(97)	5.03<br>(117)	3.96<br>(56)	3.92<br>(125)	3.11<br>(10)	2.11<br>(91)
Partner in household<br>% (n)												
yes	69.96<br>(7503)	64.77<br>(8054)	90.31<br>(3457)	88.74<br>(725)	70.15<br>(1979)	68.63<br>(1234)	54.45<br>(1273)	56.11<br>(1304)	38.16<br>(540)	38.73<br>(1234)	78.88<br>(254)	82.53<br>(3557)
no	29.56<br>(3170)	34.77<br>(4324)	9.33<br>(357)	11.14<br>(91)	29.35<br>(828)	30.81<br>(554)	45.25<br>(1058)	43.46<br>(1010)	60.78<br>(860)	60.14<br>(1916)	20.81<br>(67)	17.47<br>(753)
missing	0.48<br>(51)	0.46<br>(57)	0.37<br>(14)	0.12<br>(1)	0.50<br>(14)	0.56<br>(10)	0.30<br>(7)	0.43<br>(10)	1.06<br>(15)	1.13<br>(36)	0.31<br>(1)	0.0<br>(0)
Children <16 years in household<br>% (n)												
yes	36.30<br>(3893)	38.63<br>(4804)	66.04<br>(2528)	62.91<br>(514)	17.94<br>(506)	14.07<br>(253)	13.99<br>(327)	14.46<br>(336)	25.23<br>(357)	28.06<br>(894)	54.35<br>(175)	65.13<br>(2807)
no	63.70<br>(6831)	61.37<br>(7631)	33.96<br>(1300)	37.09<br>(303)	82.06<br>(2315)	85.93<br>(1545)	86.01<br>(2011)	85.54<br>(1988)	74.77<br>(1058)	71.94<br>(2292)	45.65<br>(147)	34.87<br>(1503)
Migration background<br>% (n)												
none	74.37	73.53	71.53	72.58	76.18	76.81	76.99	76.51	74.49	74.80	72.67	69.81

	(7975)	(9144)	(2738)	(593)	(2149)	(1381)	(1800)	(1778)	(1054)	(2383)	(234)	(3009)
direct	17.82 (1911)	18.17 (2326)	22.73 (870)	21.91 (179)	15.24 (430)	14.35 (258)	12.19 (285)	12.95 (301)	17.88 (253)	17.80 (567)	22.67 (73)	23.69 (1021)
indirect	7.81 (838)	7.76 (965)	5.75 (220)	5.51 (45)	8.58 (242)	8.84 (159)	10.82 (253)	10.54 (245)	7.63 (108)	7.41 (236)	4.66 (15)	6.50 (280)
Household language % (n)												
German	88.66 (9508)	89.31 (11106)	85.71 (3281)	87.27 (713)	89.44 (2523)	90.16 (1621)	92.34 (2159)	92.30 (2145)	89.19 (1262)	89.55 (2853)	87.89 (283)	87.56 (3774)
other	11.11 (1191)	10.39 (1292)	14.13 (541)	12.61 (103)	10.32 (291)	9.84 (177)	7.53 (176)	7.44 (173)	10.32 (146)	9.79 (312)	11.49 (37)	12.23 (527)
missing	0.23 (25)	0.30 (37)	0.16 (6)	0.12 (1)	0.25 (7)	0 (0)	0.13 (3)	0.26 (6)	0.49 (7)	0.66 (21)	0.62 (2)	0.21 (9)
Missing company % (n)												
often	37.02 (3970)	42.71 (5311)	32.55 (1246)	34.15 (279)	29.60 (835)	26.97 (485)	44.35 (1037)	47.16 (1096)	50.95 (721)	50.13 (1597)	40.68 (131)	43.02 (1854)
seldom/never	62.80 (6735)	57.15 (7107)	67.27 (2575)	65.73 (537)	70.19 (1980)	72.91 (1311)	55.47 (1297)	52.71 (1225)	48.90 (692)	49.69 (1583)	59.32 (191)	56.87 (2451)
missing	0.18 (19)	0.14 (17)	0.18 (7)	0.12 (1)	0.21 (6)	0.11 (2)	0.17 (4)	0.13 (3)	0.14 (2)	0.19 (6)	0 (0)	0.12 (5)
Disability status % (n)												
yes	11.20 (1201)	9.93 (1235)	7.52 (288)	7.34 (60)	15.10 (426)	15.74 (283)	10.69 (250)	11.79 (274)	15.34 (217)	13.03 (415)	6.21 (20)	4.71 (203)
no	88.66 (9508)	89.84 (11172)	92.48 (3540)	92.53 (756)	84.79 (2392)	84.09 (1512)	89.09 (2083)	88.08 (2047)	84.24 (1192)	86.53 (2757)	93.48 (301)	95.13 (4100)
missing	0.14 (15)	0.23 (28)	0 (0)	0.12 (1)	0.11 (3)	0.17 (3)	0.21 (5)	0.13 (3)	0.42 (6)	0.44 (14)	0.31 (1)	0.16 (7)
Urbanity/rurality % (n)												
urban	64.27 (6892)	65.40 (8132)	62.59 (2396)	62.06 (507)	64.48 (1819)	66.02 (1187)	65.01 (1520)	64.63 (1502)	66.64 (943)	65.47 (2086)	66.46 (214)	66.13 (2850)
rural	35.73 (3832)	34.60 (4303)	37.41 (1432)	37.94 (310)	35.52 (1002)	33.98 (611)	34.99 (818)	35.37 (822)	33.36 (472)	34.53 (1100)	33.54 (108)	33.87 (1460)
Household help % (n)												
yes, regularly	5.95 (638)	5.61 (697)	5.98 (229)	7.71 (63)	6.66 (188)	6.79 (122)	4.88 (114)	5.55 (129)	6.08 (86)	5.15 (164)	6.52 (21)	5.08 (219)
yes, occasionally	1.49 (160)	1.54 (192)	1.38 (53)	1.22 (10)	1.52 (43)	1.22 (22)	1.37 (32)	1.59 (37)	2.12 (30)	2.45 (78)	0.62 (2)	1.04 (45)
no	92.51 (9921)	92.81 (11541)	92.58 (3544)	91.06 (744)	91.74 (2588)	91.99 (1654)	93.76 (2192)	92.77 (2156)	91.73 (1298)	92.31 (2941)	92.86 (299)	93.87 (4046)
missing	0.05 (5)	0.04 (5)	0.05 (2)	0 (0)	0.07 (2)	0 (0)	0 (0)	0.09 (2)	0.0 (1)	0.09 (3)	0 (0)	0 (0)

Table S4: Description of men and women with no overlap in the distribution of covariables used to compute the Intersectional Gender-Score.

Variable		
N	110	
	men	women
**Sex**<br>**% (n)**	27.27 (30)	72.72 (80)
**Employment status**<br>**% (n)**		
Working and in education	0	0
full-time	100 (30)	0
part-time	0	0
some-time	0	1.25 (1)
education	0	0
retired	0	0
maternity/parental leave	0	97.5 (78)
unemployed	0	0
housewife/ -husband	0	1.25 (1)
other	0	0
missing	0	0
**Education**<br>**% (n)**		
high	0	11.25 (9)
middle	0	56.25 (45)
low	86.67 (26)	11.25 (9)
else	13.33 (4)	16.25 (13)
missing	0	5.00 (4)
**Age group**<br>**% (n)**		
18-29 years	0	16.25 (13)
30-39 years	0	73.75 (59)
40-49 years	16.67 (5)	10.00 (8)
50-59 years	66.67 (20)	0
60-69 years	16.67 (5)	0
70-70 years	0	0
70+	0	0
**Occupational status**<br>**% (n)**		
blue collar	86.67 (26)	0
white collar	3.33 (1)	0
civil servant	6.67 (2)	0
freelance	3.33 (1)	0
helping family	0	2.50 (2)
apprentice-ship	0	0
else	0	97.5 (78)
missing	0	0

**Partner in household % (n)**		
yes	0	26.25 (21)
no	100 (30)	73.75 (59)
missing	0	0
**Disability % (n)**		
yes	33.33 (10)	1.25 (1)
no	66.67 (20)	97.50 (78)
missing	0	1.25 (1)
**Children under 16 in household % (n)**		
yes	100 (30)	97.50 (78)
no	0	0.25 (2)
**Feeling lonely % (n)**		
often	56.67 (12)	17.50 (66)
seldom/never	40.0 (17)	82.50 (14)
missing	3.33 (1)	0
**Household help % (n)**		
yes, regularly	0	7.50 (6)
yes, sometimes	0	1.25 (1)
no	100 (30)	91.25 (73)
missing	0	0
**Household language % (n)**		
German	83.33 (25)	80.00 (64)
other	16.67 (5)	18.75 (15)
missing	0	1.25 (1)
**Urbanity/rurality % (n)**		
urban	86.67 (26)	52.50 (42)
rural	13.33 (4)	47.50 (38)
**Migration background % (n)**		
none	66.67 (20)	53.75 (43)
direct	23.33 (7)	35.00 (28)
indirect	10.00 (3)	11.25 (9)
